# BLOOD-BASED BIOMARKERS FOR ALZHEIMER’S DISEASE AND NEURODEGENERATION IN RELATION TO INCIDENT COGNITIVE STATUS

**DOI:** 10.1093/geroni/igae098.0003

**Published:** 2024-12-31

**Authors:** Kevin Sullivan, Chad Blackshear, James Pike, Keenan Walker, B Gwen Windham, Thomas Mosley, Michael Griswold, Priya Palta

**Affiliations:** University of Mississippi Medical Center, Jackson, Mississippi, United States; University of Mississippi Medical Center, Jackson, Mississippi, United States; New York University, New York, New York, United States; National Institute on Aging, Baltimore, Maryland, United States; University of Mississippi Medical Center - The MIND Center, Jackson, Mississippi, United States; University of Mississippi Medical Center - The MIND Center, Jackson, Mississippi, United States; University of Mississippi Medical Center - The MIND Center, Jackson, Mississippi, United States; University of North Carolina, Chapel Hill, North Carolina, United States

## Abstract

Blood-based biomarkers for Alzheimer’s Disease (AD) and neurodegeneration, including amyloid-beta (AB), ptau-181, glial fibrillary acidic protein (GFAP), and neurofilament light (NFL), could advance the monitoring and detection of individuals at high risk for adverse cognitive outcomes. At visit 5 (2011-13) of the community-based ARIC study, AB42, AB40, ptau-181, GFAP, and NFL were measured in plasma using ultrasensitive single-molecule array (Simoa; Quanterix) assays in a sample of 919 cognitively unimpaired participants (mean age 76±5 years, 61% female, 31% Black). Cognitive outcomes included adjudicated dementia and mild cognitive impairment (MCI) ascertained using a neuropsychological battery, functional status questionnaires, and informant interviews across three in-person clinic exams [median follow-up of 6.5 years (IQR:5.8-6.9)]. Multinomial logistic regressions estimated Relative Risk Ratios (RRR) of the four-category outcome of MCI, dementia, and death vs. cognitively unimpaired status for each base(2) log-transformed biomarker separately, adjusted for age, sex, education, site-race, hypertension, diabetes, obesity, APOEε4, and estimated glomerular filtration rate. All blood biomarkers were associated with incident dementia risk [AB42:40 (RRR=0.57, 95% CI: 0.40,0.83); ptau-181:AB42 (RRR=2.40, 95% CI: 1.74,3.30); GFAP (RRR=2.35, 95% CI: 1.22,4.53); NFL (RRR= 1.95, 95% CI: 1.29, 2.94)] and incident MCI risk [AB42:40 (RRR=0.77, 95% CI: 0.62,0.95); ptau-181:AB42 (RRR=1.61, 95% CI: 1.31,1.99); GFAP (RRR=1.24, 95% CI: 0.99,1.56); NFL (RRR= 1.44, 95% CI: 1.11, 1.87)]. Blood biomarkers indicative of varying neuropathological processes show potential utility in cognitive risk assessment and disease characterization in Black and White older adults with up to a decade of follow-up.

